# Supplementary material for: The frequency, risk factors and spatial distribution associated with having a diagnosis of leishmaniosis in dogs in the UK in 2019
Source: PLoS One. 2026 Mar 4;21(3):e0341610. doi: 10.1371/journal.pone.0341610 (PMC12959672; doi:10.1371/journal.pone.0341610)
Supplement: S1 Appendix — (PDF) [file pone.0341610.s001.pdf]

2021 Year Totals

	Number of consignments			Number of Animals		
Country Of Origin	Dogs	Cats	Ferrets	Dogs	Cats	Ferrets
**Austria**	10	2	1	22	2	1
**Belgium**	69	4	0	78	4	0
**Bulgaria**	856	63	0	1093	73	0
**Croatia**	364	10	0	2003	31	0
**Cyprus**	3620	337	0	3758	501	0
**Czech Republic**	203	67	0	221	77	0
**Denmark**	26	3	0	719	6	0
**England**	48	9	0	72	9	0
**Estonia**	8	1	0	23	1	0
**Finland**	8	1	0	8	2	0
**France**	140	34	0	411	44	0
**Germany**	60	40	0	76	50	0
**Greece**	739	109	0	1017	148	0
**Hungary**	3051	149	0	4517	194	0
**Isle of Man**	0	0	0	0	0	0
**Iceland**	1	0	0	1	0	0
**Ireland (Rep. of)**	3339	10	1	6658	49	12
**Italy**	271	22	0	296	33	0
**Latvia**	357	112	0	512	185	0
**Lithuania**	591	193	0	678	210	0
**Luxembourg**	2	0	0	2	0	0
**Malta**	2	2	0	3	3	0
**Netherlands**	64	11	0	152	14	0
**Northern Ireland**	2	0	0	2	0	0
**Norway**	3	1	0	3	1	0
**Poland**	4754	736	0	5594	853	0
**Portugal**	316	32	0	500	80	0
**Romania**	27212	1771	0	38081	2439	0
**Scotland**	1	2	0	1	2	0
**Slovakia**	229	19	0	291	20	0
**Slovenia**	7	2	0	7	2	0
**Spain**	4712	260	0	5909	545	0
**Sweden**	23	5	0	49	7	0
**Switzerland**	6	2	0	8	3	0
**UK**	2	0	0	1	0	0
**Total**	**51096**	**4009**	**2**	**72766**	**5588**	**13**

2022 Year Totals						
	Number of consignments			Number of Animals		
Country Of Origin	Dogs	Cats	Ferrets	Dogs	Cats	Ferrets
**Austria**	3	1	1	5	1	1
**Belgium**	20	10	0	27	15	0
**Bulgaria**	1563	123	0	2273	175	0
**Croatia**	707	33	0	2897	67	0
**Cyprus**	3025	427	0	3190	608	0
**Czech Republic**	115	25	0	137	28	0
**Denmark**	13	4	0	260	5	0
**England**	38	3	0	48	3	0
**Estonia**	16	6	0	18	6	0
**Finland**	6	0	0	6	0	0
**France**	95	23	0	123	26	0
**Germany**	79	24	0	108	31	0
**Greece**	359	61	0	431	86	0
**Hungary**	1751	103	0	2593	123	0
**Isle of Man**	1	0	0	2	0	0
**Ireland (Rep. of)**	2864	7	0	6719	11	0
**Italy**	228	15	0	289	20	0
**Latvia**	317	150	0	448	220	0
**Lithuania**	363	130	0	396	142	0
**Luxembourg**	1	0	0	1	0	0
**Malta**	1	1	0	1	3	0
**Netherlands**	54	10	1	96	12	5
**Northern Ireland**	8	1	0	39	17	0
**Norway**	6	0	0	7	0	0
**Poland**	1110	173	1	1271	218	2
**Portugal**	174	25	0	368	56	0
**Romania**	8055	653	0	10485	863	0
**Scotland**	2	0	0	3	0	0
**Slovakia**	172	18	0	181	20	0
**Slovenia**	2	0	0	2	0	0
**Spain**	3992	271	0	4845	442	0
**Sweden**	11	3	0	12	3	0
**Switzerland**	2	2	0	2	3	0
**UK**	1	0	0	1	0	0
**Total**	**25154**	**2302**	**3**	**37284**	**3204**	**8**

2023 Year Totals						
	Number of consignments			Number of Animals		
Country Of Origin	Dogs	Cats	Ferrets	Dogs	Cats	Ferrets
**Austria**	5	1	0	6	2	0
**Belgium**	8	2	0	109	2	0
**Bulgaria**	1082	100	0	1562	131	0
**Croatia**	1594	72	0	3265	140	0
**Cyprus**	2391	333	0	2598	440	0
**Czech Republic**	95	23	0	110	29	0
**Denmark**	15	0	0	405	0	0
**England**	25	4	0	26	6	0
**Estonia**	47	44	0	57	49	0
**Finland**	3	0	0	3	0	0
**France**	26	8	0	49	12	0
**Germany**	19	9	0	19	9	0
**Greece**	299	41	0	342	63	0
**Hungary**	1111	67	0	1377	94	0
**Isle of Man**	0	0	0	0	0	0
**Ireland (Rep. of)**	2839	10	3	6389	16	74
**Italy**	217	11	0	266	13	0
**Latvia**	198	110	0	268	132	0
**Lithuania**	320	77	0	544	91	0
**Luxembourg**	0	0	0	0	0	0
**Malta**	1	0	0	1	0	0
**Netherlands**	15	4	0	29	4	0
**Northern Ireland**	8	0	0	49	0	0
**Norway**	4	0	0	4	0	0
**Poland**	235	73	0	287	82	0
**Portugal**	143	12	0	293	22	0
**Romania**	10010	823	0	13184	1088	0
**Scotland**	0	0	0	0	0	0
**Slovakia**	99	12	0	105	18	0
**Slovenia**	5	0	0	5	0	0
**Spain**	2731	211	0	3333	336	0
**Sweden**	7	0	0	7	0	0
**Switzerland**	1	3	0	1	4	0
**UK**	0	0	0	0	0	0
**Total**	**23553**	**2050**	**3**	**34693**	**2783**	**74**

2024 Year Totals (01/01 to 31/05/2024)						
	Number of Consignments			Number of Animals		
Country of Origin	Dogs	Cats	Ferrets	Dogs	Cats	Ferrets
**Croatia**	669	24	0	1276	43	0
**Cyprus**	896	114	0	971	174	0
**Republic of Ireland**	1233	0	0	2597	0	0
**Italy**	101	0	0	131	0	0
**Portugal**	43	2	0	98	8	0
**Greece**	178	16	0	196	44	0
**Spain**	970	62	0	1154	129	0
**Hungary**	360	34	0	527	49	0
**Poland**	245	33	0	268	39	0
**Slovakia**	37	2	0	43	4	0
**Czechia**	32	3	0	39	3	0
**Romania**	3811	451	0	4800	535	0
**Bulgaria**	453	31	0	585	38	0
**France**	13	5	0	26	10	0
**Estonia**	40	21	0	46	24	0
**Lithuania**	151	40	0	292	47	0
**Germany**	12	4	0	15	6	0
**Netherlands**	4	0	0	5	0	0
**Latvia**	13	7	0	14	7	0
**Sweden**	1	0	0	1	0	0
**Finland**	1	0	0	1	0	0
**Belgium**	5	1	0	5	1	0
**Austria**	2	0	0	2	0	0
**Northern Ireland**	1	0	0	2	0	0
**Switzerland**	1	2	0	1	2	0
**Slovenia**	0	2	0	0	2	0
**Total**	**9272**	**854**	**0**	**13095**	**1165**	**0**

Year Totals 2021					
	Number of consignments			Number of Ani	
Country Of Origin	Dogs	Cats	Ferrets	Dogs	Cats
**Afghanistan**	0	1	0	0	1
**Albania**	0	0	0	0	0
**Algeria**	0	0	0	0	0
**Angola**	0	0	0	0	0
**Antigua and Barbuda**	0	0	0	0	0
**Argentina**	12	8	0	17	12
**Armenia**	0	0	0	0	0
**Aruba**	0	0	0	0	0
**Australia**	254	87	0	345	154
**Azerbaijan**	0	0	0	0	0
**Bahamas**	1	0	0	1	0
**Bahrain**	34	28	0	55	93
**Bangladesh**	0	0	0	0	0
**Barbados**	15	0	0	22	0
**Belarus**	1	1	0	1	1
**Belize**	0	0	0	0	0
**Bermuda**	9	3	0	12	5
**Bolivia**	0	0	0	0	0
**Bosnia and Herzegovina**	0	0	0	0	0
**Botswana**	0	0	0	0	0
**Brazil**	93	35	0	125	63
**Brunei**	0	0	0	0	0
**Cambodia**	0	0	0	0	0
**Canada**	140	87	0	167	133
**Cape Verde**	0	0	0	0	0
**Cayman Islands**	12	3	0	15	7
**Chile**	0	2	0	0	5
**China**	97	21	0	174	27
**Colombia**	12	6	0	12	7
**Costa Rica**	10	1	0	13	3
**Cuba**	0	0	0	0	0
**Curacao**	0	0	0	0	0
**Dominican Republic**	0	0	0	0	0
**Ecuador**	2	0	0	2	0
**Egypt**	20	69	0	39	436
**El Salvador**	1	0	0	2	0
**Eritrea**	0	0	0	0	0
**Ethiopia**	3	1	0	4	1
**Falkland Islands**	0	0	0	0	0

**Fiji**	0	0	0	0	0
**French Polynesia**	0	0	0	0	0
**Gambia**	0	0	0	0	0
**Georgia**	0	0	0	0	0
**Ghana**	0	0	0	0	0
**Grenada**	0	0	0	0	0
**Guam**	1	1	0	3	4
**Guatemala**	0	0	0	0	0
**Honduras**	0	0	0	0	0
**Hong Kong**	132	101	0	161	161
**India**	83	27	0	96	44
**Indonesia**	2	2	0	2	4
**Iran**	0	0	0	0	0
**Iraq**	0	0	0	0	0
**Israel**	9	10	0	12	14
**Jamaica**	2	1	0	2	1
**Japan**	10	10	0	12	17
**Jordan**	11	14	0	13	21
**Kazakhstan**	0	0	0	0	0
**Kenya**	25	13	0	43	25
**Korea (North)**	0	0	0	0	0
**Korea (South)**	51	16	0	60	19
**Kosovo**	0	0	0	0	0
**Kuwait**	18	22	0	30	44
**Kyrgyzstan**	0	0	0	0	0
**Laos**	0	0	0	0	0
**Lebanon**	22	16	0	36	29
**Liberia**	0	0	0	0	0
**Libya**	0	0	0	0	0
**Macao**	1	1	0	1	2
**Madagascar**	0	0	0	0	0
**Malawi**	2	0	0	2	0
**Malaysia**	50	37	0	71	59
**Maldives**	0	0	0	0	0
**Mauritius**	2	0	0	3	0
**Mexico**	10	6	0	11	7
**Mongolia**	0	0	0	0	0
**Morocco**	0	0	0	0	0
**Mozambique**	2	0	0	2	0
**Myanmar**	0	1	0	0	1
**Namibia**	3	1	0	3	2
**Nepal**	1	0	0	1	0
**Netherlands Antilles**	0	0	0	0	0
**New Zealand**	68	32	0	92	59
**Nigeria**	2	0	0	2	0

North Macedonia	0	0	0	0	0
Oman	7	30	0	14	72
Pakistan	0	1	0	0	1
Panama	2	1	0	2	1
Peru	19	4	0	25	6
Philippines	1	0	0	1	0
Puerto Rico	0	0	0	0	0
Qatar	58	68	0	179	239
Russia	207	196	0	261	299
Rwanda	0	0	0	0	0
Saint Kitts and Nevis	0	0	0	0	0
Saint Lucia	0	1	0	0	1
Saint Vincent and the grenadine	0	0	0	0	0
Saint Maarten	0	0	0	0	0
Saudi Arabia	18	45	0	27	82
Senegal	0	0	0	0	0
Serbia	2	0	0	2	0
Seychelles	0	0	0	0	0
Singapore	107	47	0	141	76
South Africa	887	415	1	1522	862
Sri Lanka	0	1	0	0	1
Sudan	0	0	0	0	0
Syria	0	0	0	0	0
Taiwan	4	1	0	4	1
Tajikistan	0	0	0	0	0
Tanzania	0	0	0	0	0
Thailand	41	40	0	48	48
Timor-Leste	0	0	0	0	0
Trinidad and Tobago	0	0	0	0	0
Tunisia	0	0	0	0	0
Turkey	84	8	0	148	13
Turkmenistan	0	0	0	0	0
Turks and Caicos	0	0	0	0	0
UAE	302	418	0	499	1928
Uganda	0	0	0	0	0
Ukraine	5	9	0	6	10
Uruguay	0	0	0	0	0
USA	705	267	2	966	432
Uzbekistan	0	0	0	0	0
Venezuela	0	0	0	0	0
Vietnam	2	2	0	2	3
Virgin Islands (British)	0	0	0	0	0
Zambia	0	0	0	0	0
Zimbabwe	13	5	0	22	10
**Total**	**3687**	**2223**	**3**	**5533**	**5546**

mals
Ferrets
0
0
0
0
0
0
0
0
0
0
0
0
0
0
0
0
0
0
0
0
0
0
0
0
0
0
0
0
0
0
0
0
0
0
0
0
0
0
0

Year Totals 2022				
	Number of consignments			Num
Country Of Origin	Dogs	Cats	Ferrets	Dogs
Afghanistan	0	0	0	0
Albania	1	0	0	1
Algeria	1	0	0	1
Angola	0	0	0	0
Antigua and Barbuda	1	1	0	1
Argentina	4	7	0	6
Armenia	0	0	0	0
Aruba	0	0	0	0
Australia	338	125	0	477
Azerbaijan	0	1	0	0
Bahamas	0	2	0	0
Bahrain	44	67	0	105
Bangladesh	0	0	0	0
Barbados	15	2	0	20
Belarus	0	0	0	0
Belize	0	0	0	0
Bermuda	13	5	0	16
Bolivia	0	0	0	0
Bosnia and Herzegovina	0	0	0	0
Botswana	0	0	0	0
Brazil	59	39	0	87
Brunei	1	2	0	2
Cambodia	0	0	0	0
Canada	102	70	0	141
Cape Verde	0	0	0	0
Cayman Islands	13	11	0	15
Chile	7	2	0	9
China	57	27	0	131
Colombia	11	9	0	15
Costa Rica	6	1	0	7
Cuba	0	0	0	0
Curacao	0	0	0	0
Dominican Republic	1	0	0	1
Ecuador	1	0	0	1
Egypt	11	63	0	15
El Salvador	0	0	0	0
Eritrea	0	0	0	0
Ethiopia	0	0	0	0
Falkland Islands	1	0	0	1

0		Fiji	0	0	0	0
0		French Polynesia	0	0	0	0
0		Gambia	0	1	0	0
0		Georgia	0	2	0	0
0		Ghana	0	0	0	0
0		Grenada	0	0	0	0
0		Guam	0	0	0	0
0		Guatemala	0	0	0	0
0		Honduras	31	21	0	36
0		Hong Kong	0	0	0	0
0		India	141	40	0	164
0		Indonesia	1	3	0	1
0		Iran	0	0	0	0
0		Iraq	0	0	0	0
0		Israel	7	2	0	8
0		Jamaica	0	0	0	0
0		Japan	13	5	0	16
0		Jordan	6	6	0	6
0		Kazakhstan	0	0	0	0
0		Kenya	26	5	0	41
0		Korea (North)	0	0	0	0
0		Korea (South)	46	15	0	65
0		Kosovo	0	0	0	0
0		Kuwait	6	18	0	11
0		Kyrgyzstan	0	0	0	0
0		Laos	0	0	0	0
0		Lebanon	15	7	0	20
0		Liberia	0	0	0	0
0		Libya	0	0	0	0
0		Macao	1	0	0	2
0		Madagascar	0	0	0	0
0		Malawi	0	0	0	0
0		Malaysia	34	29	0	41
0		Maldives	0	0	0	0
0		Mauritius	19	4	0	42
0		Mexico	16	6	0	24
0		Mongolia	0	0	0	0
0		Morocco	0	0	0	0
0		Mozambique	0	0	0	0
0		Myanmar	0	2	0	0
0		Namibia	5	1	0	8
0		Nepal	0	0	0	0
0		Netherlands Antilles	0	0	0	0
0		New Zealand	73	62	0	104
0		Nigeria	1	0	0	1

0
0
0
0
0
0
0
0
0
0
0
0
0
0
0
0
0
0
0
2
0
0
0
0
0
0
0
0
0
0
0
0
0
0
0
0
0
4
0
0
0
0
0
0
**6**

North Macedonia	0	0	0	0
Oman	2	6	0	2
Pakistan	0	2	0	0
Panama	1	0	0	2
Peru	3	1	0	4
Philippines	6	1	0	7
Puerto Rico	0	0	0	0
Qatar	31	42	0	82
Russia	14	33	0	29
Rwanda	0	0	0	0
Saint Kitts and Nevis	0	0	0	0
Saint Lucia	2	3	0	2
Saint Vincent and the grenadines	0	1	0	0
Saint Maarten	0	0	0	0
Saudi Arabia	22	49	0	26
Senegal	0	0	0	0
Serbia	0	0	0	0
Seychelles	0	1	0	0
Singapore	78	34	0	95
South Africa	404	193	0	771
Sri Lanka	3	0	0	4
Sudan	0	0	0	0
Syria	0	0	0	0
Taiwan	1	3	0	1
Tajikistan	0	0	0	0
Tanzania	0	0	0	0
Thailand	47	39	0	55
Timor-Leste	0	0	0	0
Trinidad and Tobago	0	0	0	0
Tunisia	0	0	0	0
Turkey	61	13	0	99
Turkmenistan	0	0	0	0
Turks and Caicos	0	0	0	0
UAE	179	254	0	337
Uganda	4	1	0	5
Ukraine	1	0	0	2
Uruguay	0	0	0	0
USA	473	198	0	672
Uzbekistan	0	0	0	0
Venezuela	0	0	0	0
Vietnam	1	3	0	1
Virgin Islands (British)	0	0	0	0
Zambia	0	0	0	0
Zimbabwe	11	7	0	29
**Total**	**2472**	**1547**	**0**	**3867**

ber of Animals	
Cats	Ferrets
0	0
0	0
0	0
0	0
6	0
15	0
0	0
0	0
220	0
1	0
4	0
219	0
0	0
4	0
0	0
0	0
6	0
0	0
0	0
0	0
71	0
3	0
0	0
109	0
0	0
15	0
3	0
39	0
13	0
2	0
0	0
0	0
0	0
0	0
464	0
0	0
0	0
0	0
0	0

Year Totals 2023

	Number of consignments		
Country Of Origin	Dogs	Cats	Ferrets
**Russia**	6	21	0
**Afghanistan**	0	0	0
**Albania**	0	0	0
**Algeria**	0	0	0
**Angola**	0	0	0
**Antigua and Barbuda**	2	0	0
**Argentina**	24	6	0
**Armenia**	0	0	0
**Aruba**	0	0	0
**Australia**	330	115	0
**Azerbaijan**	0	0	0
**Bahamas**	0	0	0
**Bahrain**	50	64	0
**Bangladesh**	0	0	0
**Barbados**	10	4	0
**Belarus**	0	0	0
**Belize**	0	0	0
**Bermuda**	9	3	0
**Bolivia**	0	0	0
**Bosnia and Herzegovina**	0	0	0
**Botswana**	1	0	0
**Brazil**	76	64	0
**Brunei**	1	0	0
**Cambodia**	0	0	0
**Canada**	85	42	0
**Cape Verde**	0	0	0
**Cayman Islands**	8	4	0
**Chile**	8	1	0
**China**	18	10	0
**Colombia**	12	5	0
**Costa Rica**	5	1	0
**Cuba**	0	0	0
**Curacao**	0	0	0
**Dominican Republic**	0	0	0
**Ecuador**	1	0	0
**Egypt**	16	50	0
**El Salvador**	1	0	0
**Eritrea**	0	0	0
**Ethiopia**	1	0	0

0	0
0	0
1	0
2	0
0	0
0	0
0	0
0	0
33	0
0	0
61	0
5	0
0	0
0	0
2	0
0	0
9	0
6	0
0	0
11	0
0	0
20	0
0	0
30	0
0	0
0	0
9	0
0	0
0	0
0	0
0	0
0	0
43	0
0	0
7	0
12	0
0	0
0	0
0	0
2	0
2	0
0	0
0	0
98	0
0	0

**Falkland Islands**	1	0	0
**Fiji**	1	0	0
**French Polynesia**	0	0	0
**Gambia**	0	0	0
**Georgia**	0	0	0
**Ghana**	1	0	0
**Grenada**	0	0	0
**Guam**	1	0	0
**Guatemala**	0	0	0
**Honduras**	0	0	0
**Hong Kong**	16	8	0
**Iceland**	0	0	0
**India**	125	46	0
**Indonesia**	0	0	0
**Iran**	0	0	0
**Iraq**	0	0	0
**Israel**	0	0	0
**Jamaica**	2	0	0
**Japan**	9	6	0
**Jordan**	4	3	0
**Kazakhstan**	0	0	0
**Kenya**	10	12	0
**Korea (North)**	0	0	0
**Korea (South)**	41	5	0
**Kosovo**	0	0	0
**Kuwait**	9	15	0
**Kyrgyzstan**	0	1	0
**Laos**	0	0	0
**Lebanon**	13	2	0
**Liberia**	0	0	0
**Libya**	0	0	0
**Macao**	0	0	0
**Madagascar**	0	0	0
**Malawi**	0	0	0
**Malaysia**	24	18	0
**Maldives**	0	0	0
**Mauritius**	11	0	0
**Mexico**	13	5	0
**Mongolia**	0	0	0
**Morocco**	0	0	0
**Mozambique**	0	0	0
**Myanmar**	0	0	0
**Namibia**	5	3	0
**Nepal**	0	0	0
**Netherlands Antilles**	0	0	0

0	0
6	0
2	0
0	0
1	0
2	0
0	0
127	0
60	0
0	0
0	0
3	0
1	0
0	0
96	0
0	0
0	0
1	0
50	0
424	0
0	0
0	0
0	0
5	0
0	0
0	0
53	0
0	0
0	0
0	0
16	0
0	0
0	0
1352	0
1	0
0	0
0	0
337	0
0	0
0	0
4	0
0	0
0	0
21	0
**4109**	**0**

**New Zealand**	78	50	0
**Nigeria**	2	0	0
**North Macedonia**	0	0	0
**Oman**	4	13	0
**Pakistan**	0	0	0
**Panama**	0	0	0
**Peru**	5	2	0
**Philippines**	0	0	0
**Puerto Rico**	0	0	0
**Qatar**	20	48	0
**Rwanda**	0	0	0
**Saint Kitts and Nevis**	0	0	0
**Saint Lucia**	2	1	0
**Saint Vincent and the grenadine**	1	0	0
**Saint Maarten**	0	0	0
**Saudi Arabia**	26	42	0
**Senegal**	0	0	0
**Serbia**	2	0	0
**Seychelles**	0	0	0
**Singapore**	43	20	0
**South Africa**	270	181	0
**Sri Lanka**	0	0	0
**Sudan**	0	0	0
**Syria**	0	0	0
**Taiwan**	5	4	0
**Tajikistan**	0	0	0
**Tanzania**	0	0	0
**Thailand**	34	22	0
**Timor-Leste**	0	0	0
**Trinidad and Tobago**	0	0	0
**Tunisia**	0	0	0
**Turkey**	65	17	0
**Turkmenistan**	0	0	0
**Turks and Caicos**	0	0	0
**UAE**	127	234	0
**Uganda**	0	0	0
**Ukraine**	0	0	0
**Uruguay**	0	0	0
**USA**	400	198	5
**Uzbekistan**	0	0	0
**Venezuela**	0	0	0
**Vietnam**	0	0	0
**Virgin Islands (British)**	0	0	0
**Zambia**	0	0	0
**Zimbabwe**	6	5	0

**Total**	**2040**	**1351**	**5**

Number of Animals		
Dogs	Cats	Ferrets
7	27	0
0	0	0
0	0	0
0	0	0
0	0	0
2	0	0
31	9	0
0	0	0
0	0	0
427	192	0
0	0	0
0	0	0
113	233	0
0	0	0
14	6	0
0	0	0
0	0	0
13	6	0
0	0	0
0	0	0
1	0	0
76	71	0
2	0	0
0	0	0
103	61	0
0	0	0
10	7	0
17	1	0
27	12	0
13	12	0
8	1	0
0	0	0
0	0	0
0	0	0
1	0	0
22	341	0
1	0	0
0	0	0
1	0	0

2024 Year Totals (01/01

	Number of Consig	
Country of Origin	Dogs	Cats
**Argentina**	7	5
**Australia**	130	49
**Brazil**	28	31
**Canada**	26	14
**China**	15	0
**Colombia**	4	3
**Costa Rica**	5	4
**Egypt**	9	16
**Hong Kong**	6	4
**India**	43	15
**Japan**	5	3
**Kenya**	10	2
**Lebanon**	5	0
**Malaysia**	10	8
**Mauritius**	15	2
**Namibia**	3	0
**New Zealand**	43	17
**Panama**	1	0
**Peru**	4	0
**Qatar**	6	11
**Saudi Arabia**	14	14
**Singapore**	12	6
**South Africa**	92	51
**South Korea**	33	3
**St Lucia**	3	0
**Trinidad and Tobago**	1	0
**Turkey**	27	9
**United Arab Emirates**	53	87
**United States of America**	131	49
**Zimbabwe**	4	3
**Barbados**	7	0
**Jordan**	1	2
**Kuwait**	5	4
**Mexico**	3	0
**Oman**	1	4
**Thailand**	8	6
**Cayman Islands**	3	1
**Chile**	2	0
**Bahrain**	7	18

2	0	0
1	0	0
0	0	0
0	0	0
0	0	0
1	0	0
0	0	0
1	0	0
0	0	0
0	0	0
18	12	0
0	0	0
132	59	0
0	0	0
0	0	0
0	0	0
0	0	0
2	0	0
12	9	0
4	6	0
0	0	0
12	24	0
0	0	0
47	5	0
0	0	0
10	44	0
0	1	0
0	0	0
19	5	0
0	0	0
0	0	0
0	0	0
0	0	0
0	0	0
33	38	0
0	0	0
15	0	0
15	8	0
0	0	0
0	0	0
0	0	0
0	0	0
9	6	0
0	0	0
0	0	0

**Ethiopia**	1	0
**Antigua and Barbuda**	0	1
**Kyrgyzstan**	0	1
**Russian Federation**	0	3
**Total**	**783**	**446**

100	82	0
2	0	0
0	0	0
4	53	0
0	0	0
0	0	0
8	3	0
0	0	0
0	0	0
46	122	0
0	0	0
0	0	0
2	5	0
2	0	0
0	0	0
39	82	0
0	0	0
2	0	0
0	0	0
48	27	0
449	357	0
0	0	0
0	0	0
0	0	0
5	4	0
0	0	0
0	0	0
43	34	0
0	0	0
0	0	0
0	0	0
100	26	0
0	0	0
0	0	0
295	1375	0
0	0	0
0	0	0
0	0	0
502	307	90
0	0	0
0	0	0
0	0	0
0	0	0
0	0	0
7	8	0

2876	3681	90

to 31/05/2024)			
gnments	Number of Animals		
Ferrets	Dogs	Cats	Ferrets
0	10	9	0
0	155	84	0
0	29	34	0
0	35	16	0
0	20	0	0
0	4	4	0
0	6	4	0
0	33	114	0
0	6	8	0
0	45	22	0
0	5	4	0
0	12	2	0
0	8	0	0
0	12	9	0
0	18	2	0
0	3	0	0
0	49	21	0
0	1	0	0
0	6	0	0
0	10	43	0
0	17	48	0
0	14	8	0
0	126	97	0
0	35	5	0
0	12	0	0
0	1	0	0
0	47	12	0
0	117	683	0
0	156	74	0
0	6	8	0
0	7	0	0
0	1	3	0
0	6	8	0
0	4	0	0
0	1	6	0
0	9	7	0
0	4	1	0
0	2	0	0
0	19	80	0

0	1	0	0
0	0	2	0
0	0	1	0
0	0	3	0
**0**	**1052**	**1422**	**0**

Animals with a Rescue/Rehoming as Purpose of Movement by Country 2021	
**Row Labels**	**Sum of Commodities Number of Animals**
Austria	2
Belgium	13
Bulgaria	820
Croatia	1936
Cyprus	3601
Czechia	29
England	36
Finland	4
France	322
Germany	11
Greece	729
Hungary	1212
Italy	170
Latvia	145
Lithuania	478
Netherlands	17
Northern Ireland	1
Poland	2112
Portugal	368
Republic of Ireland	2281
Romania	37269
Slovakia	162
Slovenia	1
Spain	5687
Sweden	4
Wales	1
**Grand Total**	**57411**

Animals with a Rescue/Rehoming as Purpose of Movement by Country 2022

Row Labels	Sum of Commodities Number of Animals
Austria	2
Belgium	2
Bulgaria	1747
Croatia	2786
Cyprus	3032
Czechia	49
England	36
France	71
Germany	10
Greece	349
Hungary	884
Italy	195
Latvia	72
Lithuania	245
Netherlands	5
Northern Ireland	39
Poland	570
Portugal	317
Republic of Irelanc	3187
Romania	10272
Slovakia	133
Spain	4579
**Grand Total**	**28582**

Animals with a Rescue/Rehoming as Purpose of Movement by Country 2023	
**Row Labels**	**Sum of Commodities Number of Animals**
Bulgaria	1164
Croatia	2987
Cyprus	2199
Czechia	35
England	19
Estonia	1
France	19
Germany	4
Greece	255
Hungary	495
Italy	218
Latvia	53
Lithuania	349
Netherlands	6
Northern Ireland	49
Poland	47
Portugal	242
Republic of Ireland	3128
Romania	11187
Slovakia	63
Slovenia	1
Spain	2926
Sweden	2
**Grand Total**	**25449**

Animals with a Rescue as Purpose of Movement by Country 2024 (01/01 to 31/05/2024)	
**Row Labels**	**Sum of Commodities Number of Animals**
Bulgaria	414
Croatia	1245
Cyprus	945
France	15
Germany	1
Greece	175
Hungary	193
Italy	103
Lithuania	155
Northern Ireland	2
Poland	175
Portugal	81
Republic of Ireland	912
Romania	4361
Spain	1137
**Grand Total**	**9914**
